# Molecular Characterization and Mapping of *Fgf21* Gene in a Foodfish Species Asian Seabass

**DOI:** 10.1371/journal.pone.0090172

**Published:** 2014-02-27

**Authors:** Le Wang, Jun Hong Xia, Xiao Jun Liu, Peng Liu, Zi Yi Wan, Gen Hua Yue

**Affiliations:** 1 Molecular Population Genetics Group, Temasek Life Sciences Laboratory, National University of Singapore, Singapore, Singapore; 2 Department of Biological Sciences, National University of Singapore, Singapore, Singapore; The Ohio State University, United States of America

## Abstract

*Fgf21* is a newly discovered fibroblast growth factor. It is typically induced by fasting and plays important roles in the regulation of glucose and lipid metabolisms and energy balance in mammals, whereas potential functions of this gene in teleosts are still unknown. We identified the *Fgf21* gene and studied its functions in Asian seabass (*Lates calcarifer*). The cDNA of the *Fgf21* encoded a protein with 206 amino acids. Analysis of DNA and amino acid sequences of *Fgf21* genes revealed that the sequences and structure of the *Fgf21* genes were highly conserved in vertebrates. Real-time PCR revealed that *Fgf21* was exclusively expressed in the intestine and kidney, which was different from the expression profiles of mammals. *Fgf21* was down-regulated under fasting, whereas it was significantly increased during the LPS challenge. Exogenous recombinant *FGF21* significantly suppressed the appetite of Asian seabass. Our data suggest that *Fgf21* plays a role in energy regulation and acute phase response in Asian seabass, and may have different functions in fish and mammals. In addition, we identified one SNP in *Fgf21*. By using this SNP, the gene was mapped on the linkage group 23, where a suggestive QTL for growth was mapped previously. Association mapping identified significant associations between *Fgf21* genotypes at the SNP and growth traits. These results not only provide important information of the functions of *Fgf21*, but also suggest that the SNP in this gene can be used as a marker in selecting fast-growing individuals of Asian seabass.

## Introduction

More than 20 members of fibroblast growth factors (FGFs) have been identified in vertebrates and were classified into three types corresponding to paracrine, intracrine and endocrine type according to their functions [1]. Among these factors, the newly discovered *Fgf21* is an evolutionary merging endocrine hormone and belongs to the FGF19 subfamily, which includes *Fgf15*/*19*, *Fgf21*and *Fgf23* [2]. In mammals, *Fgf21* is typically induced in the liver by fasting and plays an important role in glucose and lipid metabolisms, ketogenesis, growth hormone signaling pathways in hepatocytes and metabolic torpor, a short-term hibernation-like state for energy conservation [1]–[6]. Studies in mice and humans demonstrated that *Fgf21* had the potential of preventing diet-induced obesity and metabolic disorders in obese cases [7]–[10]. It was suggested that *Fgf21* showed a characteristic of nutritional adaptation in terms of fasting and feeding scales [10]. Some other studies demonstrated that *Fgf21* was involved in cold-induced thermogenesis [11]. A recent study in mice revealed that the *Fgf21* was induced by acute inflammatory simulation and protected the hosts from toxic effects during acute phase response (APR) [3]. Recently, genetic association studies in humans demonstrated that common polymorphisms in the *Fgf21* signaling pathways were associated with metabolic risks and syndromes, such as high cholesterol and body mass risks [12], obesity and diabetes [13] and even dietary macronutrient intake [14]. To date, there is little information about the functions of *Fgf21* in fish [15], although cDNA sequences of several fish species (e.g. tilapia and fugu) were deposited in Genbank.

The Asian seabass (*Lates calcarifer*) belonging to the Latidae family, is an important food fish species in Southeast Asia and Australia, and has been cultured for more than 30 years [16]. Since 1998, we have conducted a selective breeding program to improve growth [17]–[20], disease resistance [21] and meat quality [22] of this species. Some candidate genes (e.g., *prolactin* and *IFABP-a* genes) related to quick growth have been identified using candidate gene methods [20] and QTL mapping [19]. Since *Fgf21* plays an important role in regulating energy balance, obesity and body weight in mammals [12], [13], we speculate that this gene may also play a role in glucose and lipid metabolisms, and even in controlling growth and body weight in fish. To test this hypothesis, we cloned the full-length cDNA, amplified its complete genomic sequence, and analyzed its expressions in different tissues under normal, fasting and acute phase response conditions. We found that the gene was exclusively expressed in the kidney and intestine, and responded to the fasting and acute phase response conditions. In addition, we identified a SNP in this gene and found significant associations between genotypes at the SNP and growth traits. Our data suggest that the SNP associated with growth can be used as a marker for marker-assisted breeding for quick growth in Asian seabass.

## Materials and Methods

### Ethics statement

All handling of fishes was conducted in accordance with the guidelines on the care and use of animals for scientific purposes set up by the Institutional Animal Care and Use Committee (IACUC) of the Temasek Life Sciences Laboratory, Singapore. The IACUC has specially approved this study within the project “Breeding of Asian seabass” (approval number is TLL (F)-12-004).

### Analysis of cDNA, genomic DNA and amino acid sequences of *Fgf21* in Asian seabass

The full length of *Fgf21* cDNA sequence was derived from our intestine transcriptome data sets of Asian seabass sequenced by Roche 454 GS FLX+ platform (NCBI SRA accession number: DRR002185-DRR002190) [21].

To amplify the genomic DNA sequence of the *Fgf21* gene, DNA was extracted from caudal fin according to the protocol developed previously [23]. Genomic sequence was amplified using the following primer sets, Lca_Fgf21F: 5′-TGTTTTTATTTCCATGCACATCTT-3′ and Lca_Fgf21R: 5′-AATTTAGGCCCATTCCAAGGAG-3′. PCR was performed on a PTC-100 thermocycler (MJ Research, CA, USA) in 25 µl volume containing 1× PCR mix buffer (Finnzymes, Vantaa, Finland), 100 nM of each primer, 50 µM of dNTP, 50 ng genomic DNA template and 1U DNA polymerase (Finnzymes, Vantaa, Finland) with the program: 94°C for 5 min, followed by 35 cycles of 94°C for 30 s, 56°C for 30 s and 72°C for 2 min and a final extension of 72°C for 5 min. PCR products were examined on 2.0% agarose gel and were further purified and sequenced on an ABI 3730xl DNA sequencer (Applied Biosystems, CA, USA).

The protein sequence of Asian seabass *Fgf21* was translated from cDNA using DnaSP v5 [24] and the protein structure was predicted by SWISS-MODEL server [25]. Putative signal peptide was estimated using SignalP 3.0 (http://www.cbs.dtu.dk/services/SignalP/). Multiple sequence alignment and phylogenetic analysis were conducted using ClustalX [26] and MrBayes 3.2 [27], respectively. Before constructing Bayesian phylogeny, we examined the amino acid mutation mode using Modeltest v3.7 [28].

### Analysis of tissue distribution of *Fgf21* with qRT-PCR

Tissue samples including brain, spleen, liver, eye, gill, heart, kidney, adipose tissue, muscle and intestine of three individuals at 9 months of age were collected and preserved in Trizol (Invitrogen, CA, USA), and then immediately stored at −80°C until RNA extraction. Total RNA was isolated using Trizol kit (Invitrogen, CA, USA). RNA purity and concentration were determined by spectrophotometry using Nanodrop 1000 (Thermo Scientific, MA, USA). The quality of the isolated RNA was checked by electrophoresis on 2.0% agarose gel. Two micrograms of DNase-treated RNA were synthesized to the first strand of cDNA using Reverse Transcriptase M-MLV (Promega, WI, USA). cDNA products were then used as template to examine the tissue distribution pattern of the *Fgf21* gene using quantitative real-time PCR (qPCR). Briefly, PCR reactions in triplicates were performed using the KAPA™ SYBR® FAST qPCR Kits (KapaBiosystems, Boston, USA) according to the manufacturer's protocol and in an iQ™5 Real Time PCR Detection Systems (Bio-Rad, CA, USA). The primer sets for *Fgf21* amplification were as follows, forward primer: 5′-GGGCATCTCTACACAGATAACCAC-3′ and reverse primer: 5′-CAGAGAAACAGGGATGACGA-3′ with the forward primer bridging the first and the second exon. A melting curve method of the amplified products was conducted to confirm the applicability of the qPCR. The 2^−ΔΔCT^ method [29] was used for analysis of the relative gene expression, and the housekeeping gene elongation factor-1 alpha (*EF1A*) was employed to normalize the relative expression level of the *Fgf21* gene in different tissues (EF1A_F: 5′-GGGCATCTCTACACAGATAACCAC-3′ and EF1A_R: 5′-CAGAGAAACAGGGATGACGA-3′) [21].

### Analysis of expression patterns of *Fgf21* during fasting and challenge with lipopolysaccharides (LPS)

Seabass juveniles at the age of 50 days post hatch (dph) with an average body weight of 5.34±0.45 g were chosen and cultured in freshwater at 25°C at the fish facility of Temasek Life Sciences Laboratory, Singapore. In the fasting challenge experiment, individuals were divided into eight groups and cultured in 12-L tanks (N = 12/group). These groups were cultured under identical conditions. Before the start of the trial, fish were fed twice daily with commercial fish feed at 9 am and 6 pm, respectively. During the period of the fasting, four groups were randomly assigned as controls and treatments. The controls were fed as usual, while the treatments were not exposed to food. Tissue samples including intestine and kidney were collected for RNA isolation at the time day 0, 3, 7 and 14, 4 hours after fasting, which covered the short-term and long-term fasting regimes.

Lipopolysaccharides (LPS) (Sigma-Aldrich, MO, USA) were used to induce acute phase response to study the response of *Fgf21*. Individuals of the same size as the above were randomly divided into two groups (30/group) and were intraperitoneally injected with 50 µl PBS and 50 µl LPS of 500 µg/ml diluted in PBS, respectively. Such dose of LPS was typically used to induce acute phase response, especially in studies of investigating the expression of *Fgf21* gene [3]. Kidney and intestine were collected at 4 h, 8 h and 24 h post challenge. The number of collected individuals for each group at each time point was from four to six.

RNA isolation and cDNA synthesis were performed as described above. Quantitative real-time (qPCR) was also carried out to investigate the expression level of *Fgf21* in different conditions as described above and the relative expression levels were calculated using 2^−ΔΔCT^ method [29]. Quantitative data were expressed as mean ± SE and statistical comparisons were performed using two-way analysis of variance (ANOVA) at the significance level of 0.05.

### Studying the effect of human recombinant *FGF21* on feed intake

Asian seabass individuals with body weight of 7.80±0.49 g were used for this study. 60 seabass were randomly divided into 10 groups with each consisting of six individuals and were cultured in 60-liter tanks under identical conditions at the fish facility of Temasek Life Sciences Laboratory, Singapore. Fish were fed with usual commercial fish feed (Biomar, Nersac, France) once daily at 9:30 am. This process lasted for three weeks so that the fish were trained to develop a regular eating schedule. Then the fish were not exposed to feed for three days before the start of the experiment. Human recombinant *FGF21* (ProSpec Protein Specialists, NJ, USA) was dissolved and diluted to 5 mg/ml in PBS (pH 7.4). At 7:30 am on the day of the experiment, seabass were anesthetized using AQUI-S® (AQUI-S, Lower Hutt, New Zealand). Five groups were randomly selected to be intraperitoneally injected with human recombinant *FGF21* solutions at a dose of 50 ng/g body weight using a 25G needle, while the other groups were injected with a corresponding volume of PBS as controls. Fish were then allowed to recover for 2 hours until the regular feeding time. At 9:30 am, slightly excessive commercial feed pellet (Biomar, Nersac, France) was provided to each seabass group. Before this trial, the feed pellets were artificially selected to have the same size and weight. Feeding was measured at 11:30 am four hours post injection, when, according to studies in mammals, the maximal effects of *FGF21* would be still sustained [30]. Consumption of feed pellets was calculated by subtracting the number of left pellets from the total number of pellets provided for each group. Group feeding was then normalized as the percentage of body weight for comparison between experimental groups and controls using *t*-test.

### Detecting a SNP in *Fgf*21 and linkage mapping of *Fgf21*

Twelve seabass individuals from different families were used to detect SNPs. In brief, whole *Fgf21* genomic sequences were targeted for sequencing using the primer set: Lca_Fgf21F and Lca_Fgf21R. The obtained sequences were aligned using Sequencher (GeneCodes, MI, USA). We also cloned the 5′ upstream sequence of *Fgf21* gene using the following primer sets (forward primer: 5′-TTTCCAAATGTTTTTGTTTC-3′ and reverse primer: 5′-TCATCTGCAGATACATCCCTGC-3′) designed according to sequence homology between European seabass (*Dicentrarchus labrax*; GenBank accession no. FQ310507) and orange-spotted grouper genome (*Epinephelus coioides*; unpublished data). Genetic variations among sequences were detected by alignment. SNPs were genotyped using Sanger sequencing with ABI 3730xl DNA sequencer (Applied Biosystems, CA, USA). Linkage mapping was based on a F_2_ reference family consisting of 359 full-sib progeny [19]. The identified SNP was analyzed to map the gene using the methods as described by Xia et al. [19] and a logarithm of odds (LOD) threshold of 3.0 was used to generate the genetic linkage map.

### QTL and association mapping for growth and fatty acid traits

The associations between genotypes and phenotype traits were examined using the same mapping family as above. The phenotypes included body weight, total length and standard length at six months and nine months [19]. Considering *Fgf21's* main functions in glucose and lipid metabolisms, we also analyzed the phenotypes of different types of fatty acid contents reported previously by our group [22]. Combining our previously analyzed markers [22] in this mapping family, QTL mapping was performed according to the methods of Xia et al. [19], [22] using the software MapQTL 4.0 [31]. In brief, QTL analyses were conducted under multiple QTL mapping model. LOD scores were obtained by bootstrapping with 10,000 permutations. QTLs with LOD threshold of more than 95% (*p*<0.05) at chromosome level and of more than 99% (*p*<0.01) at whole genome level were considered as suggestive and significant, respectively. Besides, the associations between phenotype values and genotypes at the SNP in *Fgf21* were tested using *t*-test.

## Results and Discussion

### *Fgf21* sequence characterization and phylogeny

**Figure 1:**
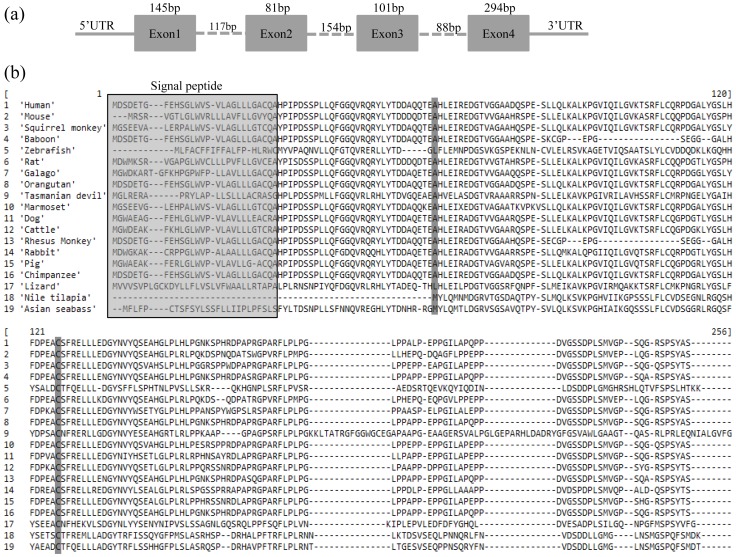
Genomic structure (a) and deduced amino acid sequence alignment (b) of the *Fgf21* gene in Asian seabass. Marked columns denoted the corresponding position of conserved cysteine residues across FGF family. Sequences used for alignment were shown in supporting information Table S1.

**Figure 2:**
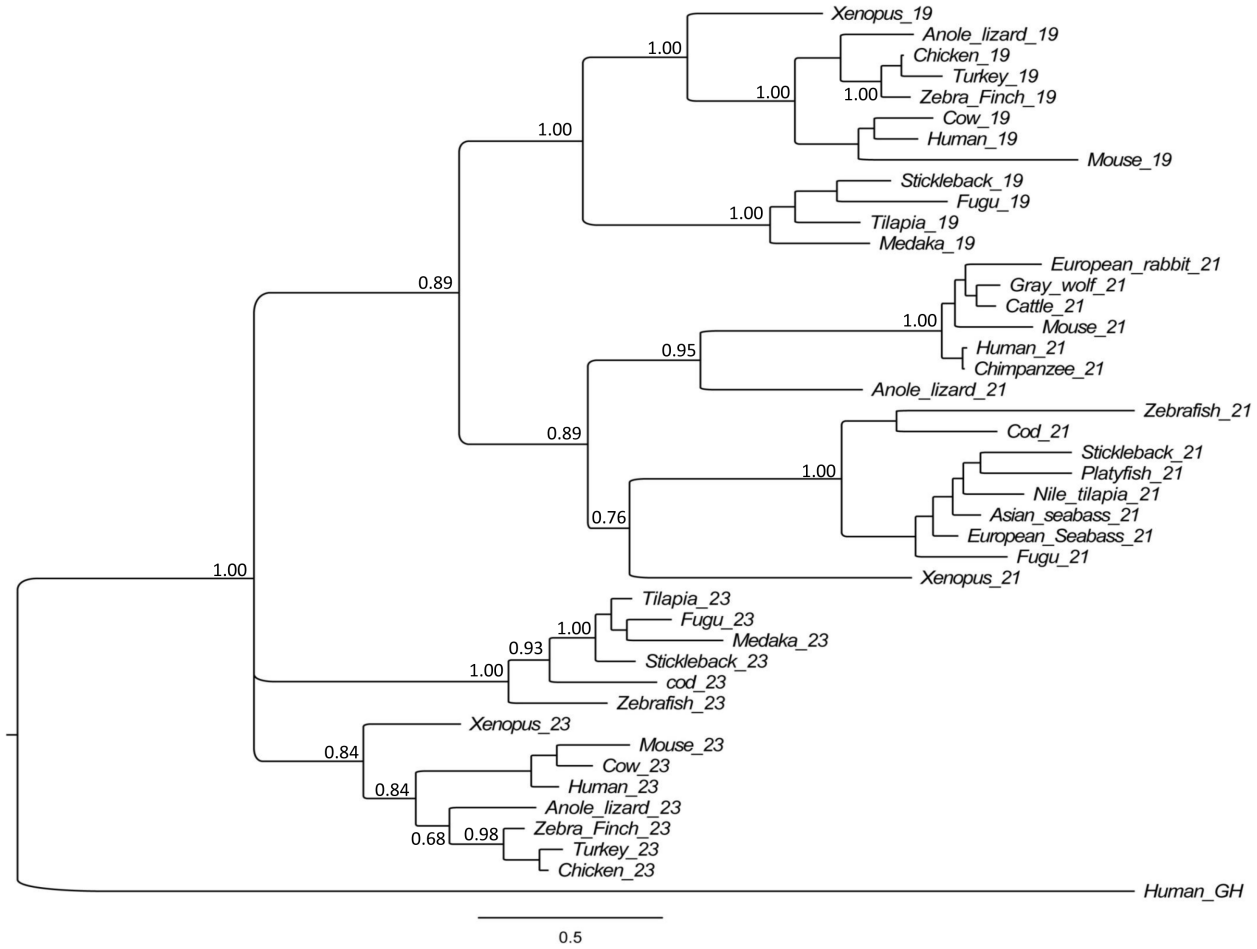
Bayesian phylogenetic relationships of *Fgf21* proteins among vertebrates, in which human growth hormone was used as outgroup. Sequences used for phylogeny construction were shown in supporting information Table S2.

The cDNA sequence *Fgf21* with a length of 912 bp containing an open reading frame (ORF) of 618 bp (Figure 1) was identified from transcriptome data (XP_003438516.1). The ORF encoded a protein with 206-amino acids (Figure 1). The gene consisted of four exons and three introns. The same genomic structure of the *Fgf21* gene was also reported in mice [32]. Protein sequences alignment among vertebrates *Fgf21* showed high levels of sequence similarity ranging from 93.3% with Nile tilapia to 59.1% with baboon (Figure 1). With comparison to model species, *FGF21* of Asian seabass showed a similarity of 65.7%, 65.6% and 69.7% to that of human, mouse and zebrafish. However, the signal peptide sequences between fish species and mammals showed much difference, suggesting that the functions of *Fgf21* proteins may be somewhat different between mammals and fish. Across the FGF family, there were two conserved cysteine residues corresponding to position 60 and 122 of human *FGF21* protein [32]. However, the conserved cysteine residues at position 60 of *FGF21* protein for the formation of disulfide bridges in the peptide sequences was substituted, which was the same in humans [32]. The cysteine residue was replaced by methionine in fish species. It was different in mammals, where it was replaced by alanine (Figure 1). *Fgf21* protein modeling for three-dimensional (3D) structure also revealed high levels of similarity of tertiary structure between Asian seabass and human except for the signal peptide region (Figure S1). We constructed a Bayesian phylogeny of the members of FGF19 subfamily including *Fgf19*, *Fgf21* and *Fgf23* among vertebrates, where mammals, reptiles, amphibians and fishes all formed their own clusters (Figure 2). The Asian seabass *Fgf21* protein showed the closest relationship with the European seabass with Bayesian posterior probability of 0.77 and significantly divergent from that of zebrafish.

### Tissue distribution and expression patterns of *Fgf21*

**Figure 3.Tissue:**
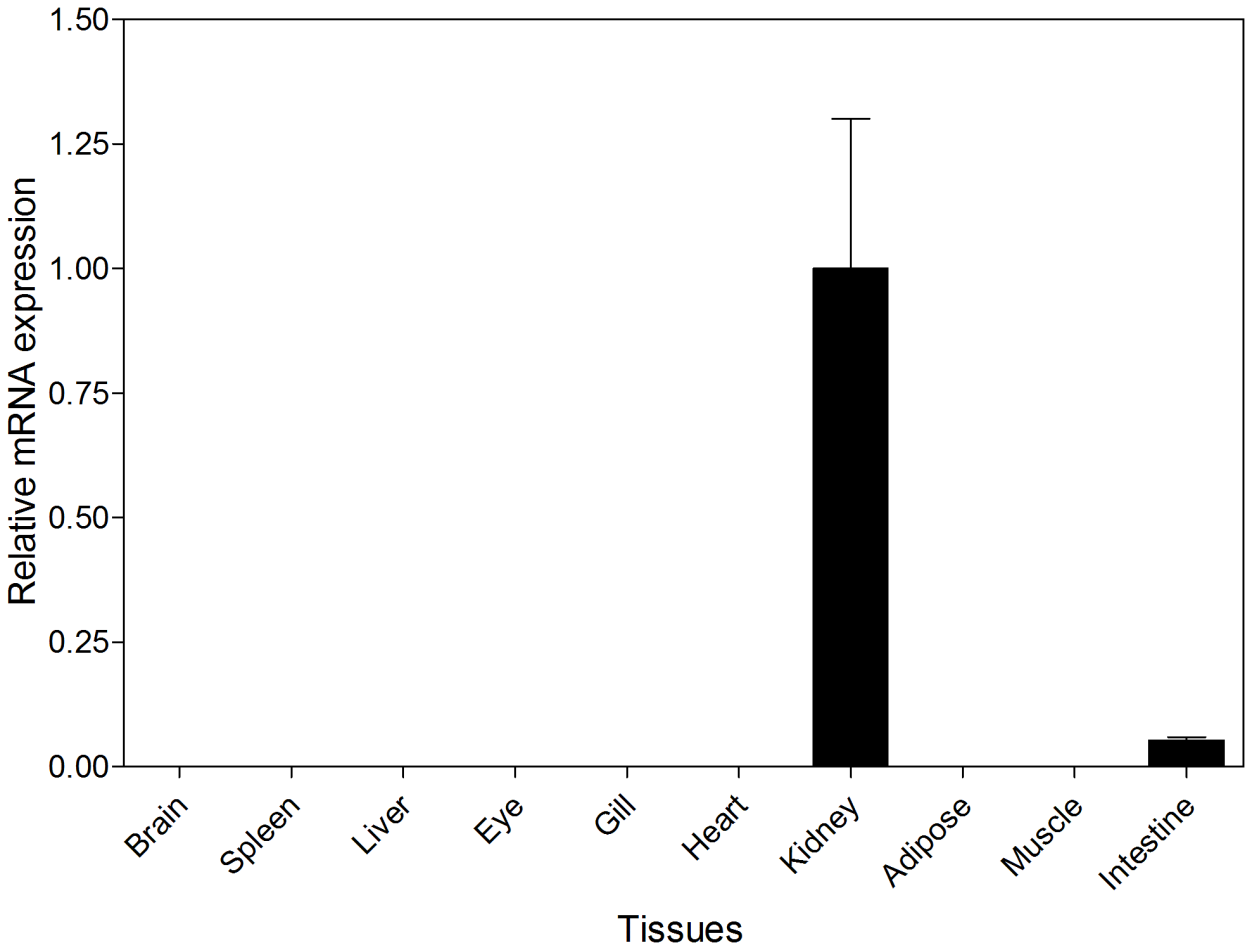
distribution of *Fgf21* mRNA revealed by real-time PCR based on three nine-month-old individuals. Each bar indicates the mean ± SE.

qPCR based on three nine-month-old seabass individuals revealed that *Fgf21* mRNA was exclusively expressed in the kidney and intestine (Figure 3). Such expression patterns were substantially different from that in mammals, where *Fgf21* was expressed in thymus, adipose tissues and preferentially in liver [32]–[36]. This result suggests that *Fgf21* proteins have different functions in fish species and mammals.

**Figure 4:**
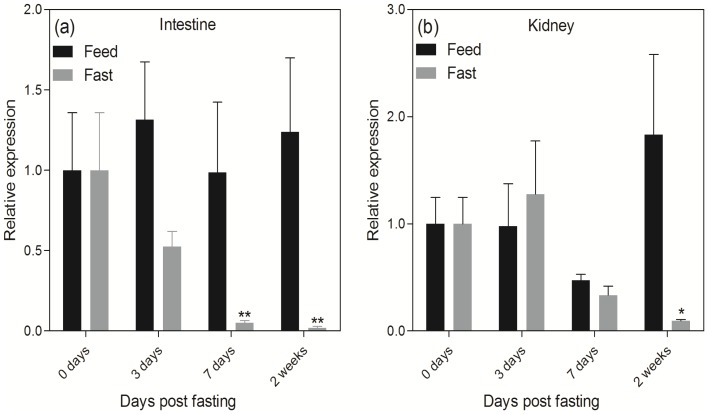
Relative expression of *Fgf21* mRNA under feeding and fasting regimes in the intestine (a) and kidney (b) quantified by real-time PCR. Each group consisted of six individuals. The data was expressed relative to that of 0 days. Statistics were calculated at the same time point between feed and fast groups. Each bar indicates the mean ± SE, * denotes *p*<0.05 and ** denotes *p*<0.01.

In order to understand the potential physiological functions of *Fgf21* in Asian seabass and examine if *Fgf21* protein shows the same functions as in the mammals, we examined its expression patterns under fasting and feeding regimes in the kidney and intestine. *Fgf21* mRNA expression levels were not significantly different between feeding and fasting groups in the short term (three days after fasting) in the two tissues (*p*>0.05, Figure 4). In the long term (i.e. after one week of fasting), however, *Fgf21* mRNA levels were significantly down-regulated especially after fasting for two weeks (*p*<0.01 in intestine and *p*<0.05 in kidney; Figure 4). In addition, we found that the down-regulation of *Fgf21* mRNA expression in intestine was significant under fasting challenge for the 7 days, whereas the down-regulation of *Fgf21* mRNA in kidney became significant after fasting for two weeks. These results suggest that *Fgf21* mRNA expression in intestine is more sensitive than in kidney under fasting challenge in Asian seabass (Figure 4). Such observation is substantially different from the results in mammals, where *Fgf21* was significantly up-regulated or induced mainly in liver and adipose tissues by fasting [33], [34], [37]. Some other studies in mammals also reported that energy utilization activities such as thermogenic activation and adrenergic stimulation significantly induced *Fgf21* mRNA expression [38]–[40]. Our results in Asian seabass directly contradicted with that of the studies in mammals, which strongly suggests different physiological functions between *Fgf21* proteins of Asian seabass and mammals. Nevertheless, further studies on the mechanism underlying *Fgf21* functions in fish species are needed to elucidate the different expression patterns between Asian seabass and mammals.

**Figure 5:**
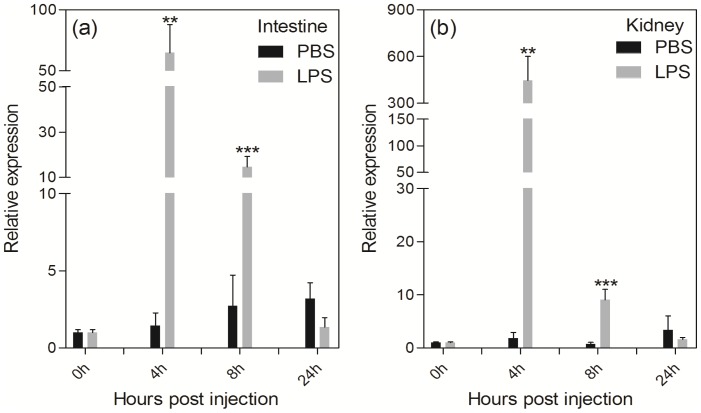
Relative expressions of *Fgf21* mRNA at different time points post challenge with PBS and LPS in the intestine (a) and kidney (b) revealed by real-time PCR, where *EF1A* was used as reference. Each group included four to six individuals. The data was expressed relative to that of 0± SE, * denotes *p*<0.05, ** denotes *p*<0.01 and *** denotes *p*<0.001.

We further checked *Fgf21* mRNA levels in the intestine and kidney upon LPS challenge. A dose of 5 µg/g body weight was used for injection. According to previous studies both on fish species and mammals, this dose of LPS was commonly used to induce acute phase response [3]. *Fgf21* mRNA levels were significantly increased in both tissues during the short term acute immune response phase at four and eight hours' time points after the LPS injection (*p*<0.01; Figure 5). However, after this acute response phase, *Fgf21* mRNA expression levels were significantly decreased to normal levels (Figure 5). We detected little difference in sensitivity between the intestine and kidney upon LPS challenge (Figure 5). These results are consistent with a previous study in mice where *Fgf21* protein was induced by inflammatory stimuli and protected from the toxic effects of lipolysis as a positive acute phase response protein [3]. In addition, *Fgf21* was also suggested to have similar functions of *leptin* in balancing glucose and fatty acid metabolism during acute phase response [3].

Altogether, the above results suggest the functions of *Fgf21* in fish species are not the same as those in mammals. The *Fgf21* gene could have lost the functions in regulating energy metabolism during long-term fasting in fish species but was retained in mammals. Differences of gene functions are common for vertebrates following the whole genome duplication and functional divergence during genes evolution [41], [42]. The decreased level of *Fgf21* mRNA is likely an adaptive response against long-term fasting. On the other hand, significant up-regulation of *Fgf21* mRNA during LPS challenge suggest this gene still possesses the same functions in regulating glucose and lipid metabolism during acute phase response in fish species as in the mammals.

### Effect of recombinant human *FGF21* on feed intake

**Figure 6:**
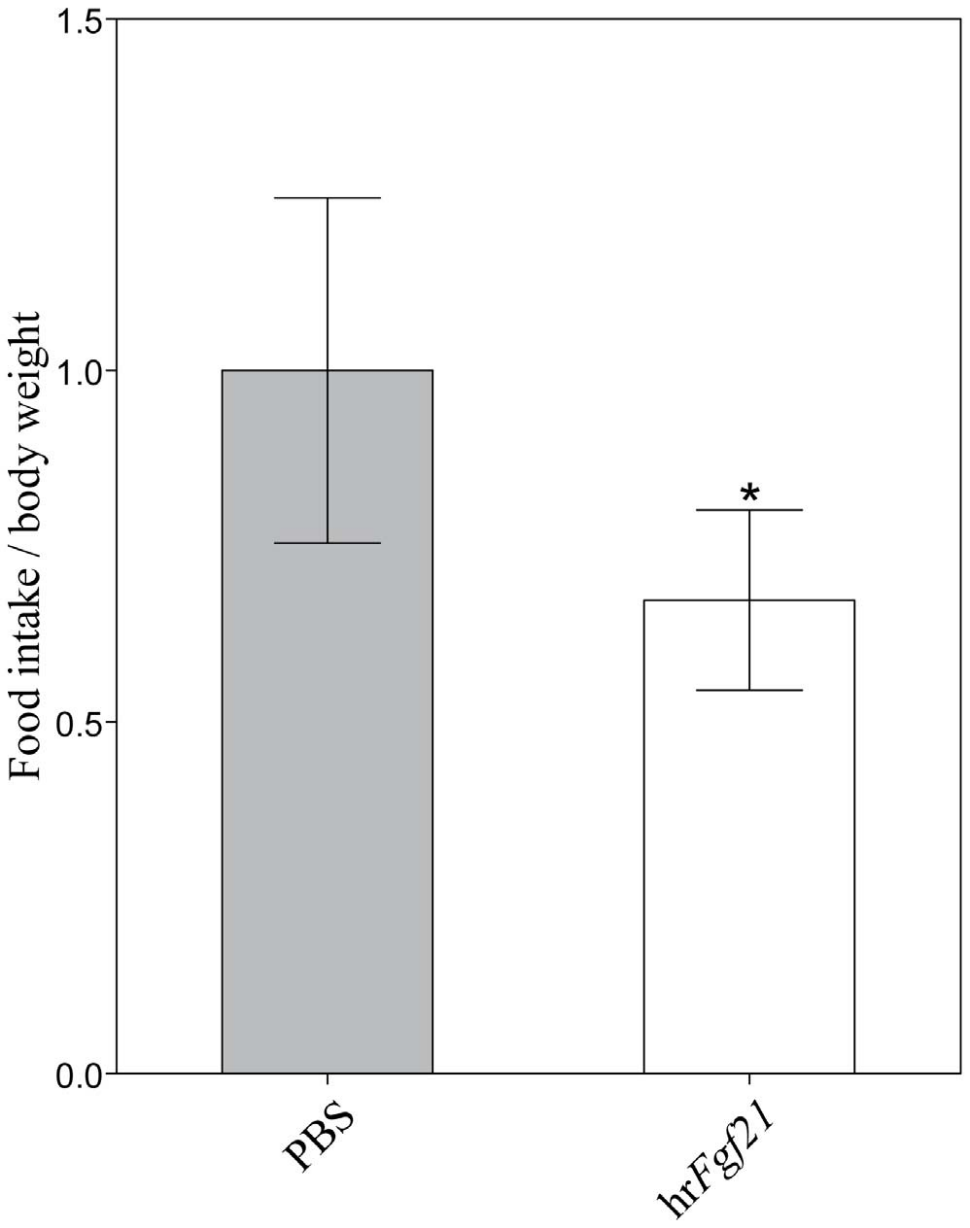
Effect of human recombinant *FGF21* (hr*FGF21*) protein (PBS as control) on appetite of Asian seabass at four hours post injection. The appetite was expressed as the ratio of total body weight to the total feed intake for each group that consisted of six individuals. Each bar indicates the mean ± SE and * denotes *p*<0.05.

It is well known that *Fgf21* has the potential function of inducing energy production by fatty acid oxidation, ketogenesis and lipolysis in mammals [33], [43]–[47]. Many studies revealed that exogenous human recombinant proteins of conserved peptide structures play roles in teleosts [48]–[50]. Protein modeling study showed that the peptide structure of *FGF21* was highly conserved between human and Asian seabass (Figure S1). Here, we examined the effects of exogenous human recombinant *FGF21* protein on seabass appetite. Previous studies revealed that a wide range of dose of recombinant human proteins, such as *LEPTIN* with a dose ranging from100 ng/g to 1 µg/g body weight, showed physiological functions in teleosts upon injection [48]–[50]. In particular, lower dose of recombinant protein (100 ng/g body weight) showed more significant effects of suppressing appetite on experiment groups [49]. Considering the similar molecular weight between *FGF21* and *LEPTIN* and the rather small size of individuals in this study (∼5 g) compared to that of the other studies (∼30 g), we therefore used a dose of 50 ng/g body weight of recombinant *FGF21* in this study. By calculating food intake at four hours post injection, we found that recombinant human *FGF21* significantly suppressed feed intake of the experimental groups of Asian seabass by 32.6% compared to the control groups (*p*<0.05; Figure 6). Such suppression of feed intake in Asian seabass could be caused by the functions of *Fgf21* in inducing energy production, which is similar to that in the mammals. Studies in mice revealed that injection of recombinant *FGF21* protein enhanced oxidative capacity and energy production [51] and reduced body weight and whole-body fat mass [45]. Altogether, our results suggest that *Fgf21* in Asian seabass have functions in inducing energy production.

### Linkage, QTL and association mapping of *Fgf21*

DNA sequence alignment among seabass individuals from different families revealed one SNP (A/G, 793) in the genomic region. This SNP was located in the fourth exon, but was a synonymous substitution (Figure S2). This mutation was then used for linkage mapping in the reference family. The *Fgf21* gene was mapped into the linkage group 23 (LG23) between marker LcaTe0399 and LcaTe0471 (Figure S2) [22]. In previous studies on QTL mapping for growth [19] and fatty acid profile [22] in an F_2_ Asian seabass by our group, significant and suggestive QTLs were separately mapped into the LG23. In the same mapping family, two suggestive QTLs for six months' body weight and the content of C18∶2 unsaturated fatty acids at chromosome level (*p*<0.05) were mapped between LcaTe0399 and LcaTe0471 on the linkage group 23. Since the *Fgf21* was located in the QTL, we conducted an association study using the F_2_ family. We detected significant relationships between six months' body weight, nine months' body weight and nine months' total length and genotypes of *Fgf21* SNP (*p*<0.05; Table 1). In another F_2_ family consisting of 32 seabass individuals, we also detected a significant association between genotypes at *Fgf21* gene SNP and bodyweight. In this family, the average body weight with GG genotypes of 51.7±16.5 g (n = 15) was significantly higher than those with the AG genotypes with an average body weight of 38.7±11.5 g (n = 17) at the age of three months post hatch (*p*<0.05). Therefore our results indicate that *Fgf21* polymorphism is associated with body weight in Asian seabass. However, we did not detect any associations between genotypes at *Fgf21* SNP and traits related to fatty acids. These results suggest that *Fgf21* gene is located near or on the intervals of the QTLs for fatty acid component profile in Asian seabass, but not responsible for these traits. Therefore, the SNP in the *Fgf21* gene can be used as a marker for growth traits in marker-assisted breeding programs of Asian seabass.

**Table 1:** Summary statistics for growth traits and fatty acid components in the Asian seabass individuals with different genotypes at the SNP (A/G) in *Fgf21*.

	Traits		Number of individuals		*p* value
Genotypes	GG	AG	GG	AG	
6BW (g)	232.7±5.1	218.1±5.0	141	137	**0.04**
6SL (cm)	207.3±1.7	203.3±1.6	141	137	0.08
6TL (cm)	239.8±1.8	237.0±1.8	141	137	0.27
9BW(g)	342.7±9.1	316.5±8.5	130	130	**0.04**
9SL (cm)	229.9±1.9	224.2±2.1	130	130	0.05
9TL (cm)	277.5±2.2	271.1±2.2	130	130	**0.04**
C14∶0 (%)	3.04±0.08	2.96±0.08	131	131	0.46
C16∶0 (%)	19.74±0.11	19.76±0.12	132	133	0.9
C16∶1 (%)	3.51±0.08	3.34±0.09	131	129	0.17
C18∶0 (%)	7.21±0.11	7.24±0.11	131	131	0.84
C18∶1 (%)	21.78±0.21	21.69±0.18	132	132	0.76
C18∶2 (%)	8.97±0.10	8.96±0.10	132	132	0.93
C18∶3 (%)	1.70±0.05	1.70±0.05	131	128	0.95
C20∶3 (%)	1.56±0.06	1.54±0.06	121	122	0.82
C20∶5 (%)	7.21±0.07	7.22±0.08	132	133	0.96
C22∶1 (%)	2.54±0.07	2.59±0.07	127	127	0.62
C22∶5 (%)	4.33±0.03	4.32±0.02	132	132	0.87
C22∶6 (%)	16.40±0.26	16.66±0.26	132	133	0.47

6BW, six months' body weight; 6SL, six months' standard length; 6TL, six months' total length; 9BW, nine months' body weight; 9SL, nine months' standard length; 9TL, nine months' total length. The values of weight and length were shown with standard errors (SE). The value of each fatty acid was reported as the percentage of the total measured fatty acids with SE [22]. Significant statistics were denoted with bold.

## Conclusion

We identified and characterized the *Fgf21* gene in Asian seabass. This is the first report on *Fgf21* in a foodfish species. The expression patterns of *Fgf21* were much different between Asian seabass and mammals, suggesting that the *Fgf21* gene has some different functions in fish and mammals. The physiological functions in regulating energy balance and suppressing appetite seemed to be similar between mammals and fish species. In addition, we found one SNP in the *Fgf21* gene and mapped the gene on the linkage group 23. QTL mapping and association analysis revealed a significant association between the SNP and growth traits. The SNP associated with growth traits may be used as a marker for selecting quick growing Asian seabass at fingerling stage. To understand more about the mechanisms underlying the role of *Fgf21* in growth and acute stress response, it is essential to further study signaling pathways involving this gene.

## Supporting Information

Figure S1 Protein 3-D structure modeling of *FGF21* protein in Asian seabass and human. The reliability of prediction was shown with different colors. Blue denoted with the highest level of reliability, while red denoted the lowest level of reliability.
(TIF)

Figure S2 Single nucleotide polymorphism in the *Fgf21* gene (a) and mapping of the *Fgf21* gene on linkage group 23 (LG23; b) of Asian seabass.
(TIF)

Table S1 Accession numbers of sequences used for alignment.
(XLSX)

Table S2 Accession numbers of sequences used for Bayesian phylogeny construction.
(XLSX)
