# Supplementary figures and images for: Molecular Characterization and Mapping of Fgf21 Gene in a Foodfish Species Asian Seabass
Source: PLoS One. 2014 Feb 27;9(2):e90172. doi: 10.1371/journal.pone.0090172 (PMC3937445; doi:10.1371/journal.pone.0090172)

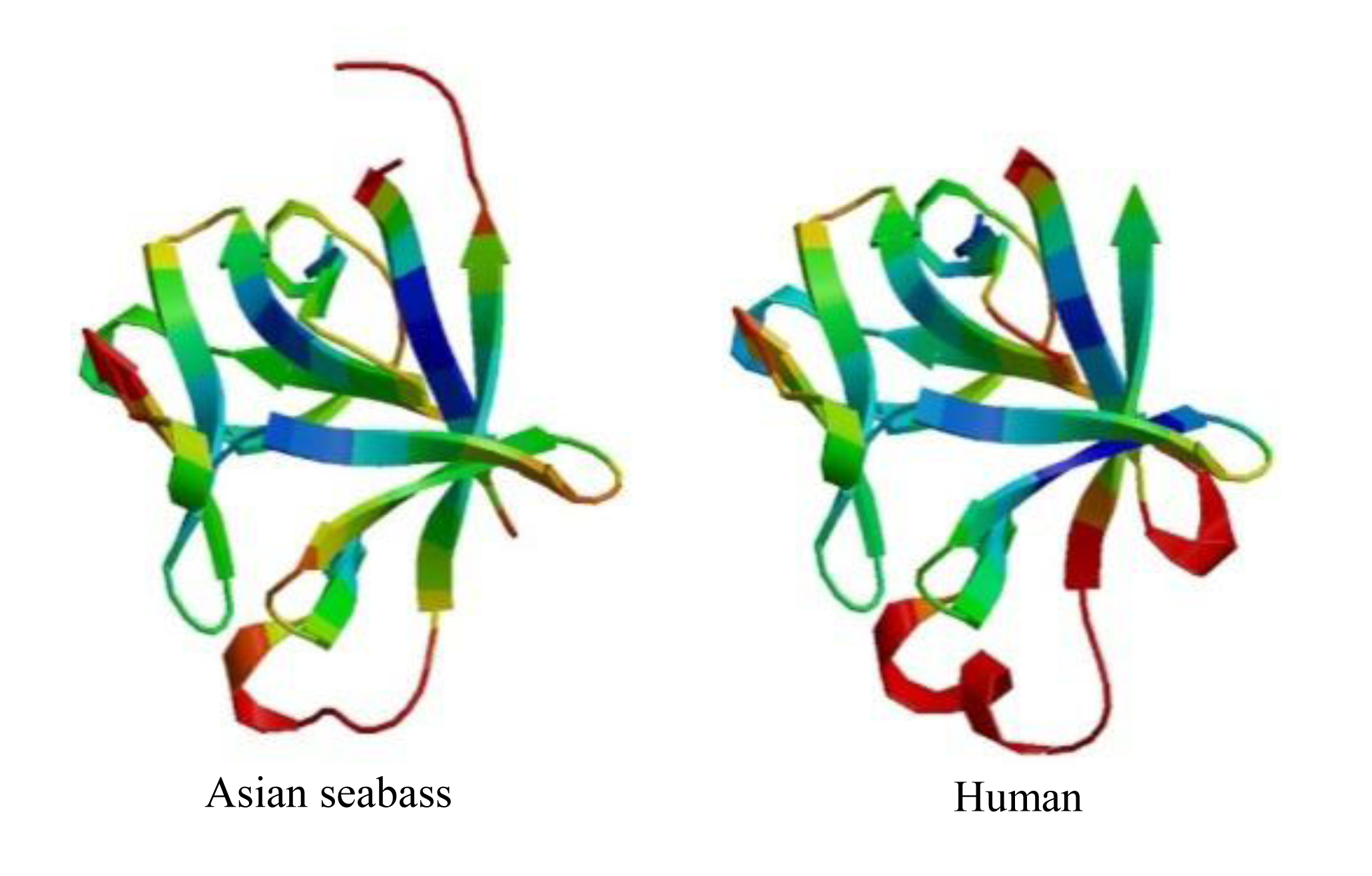

Supplement: Figure S1 — Protein 3-D structure modeling of FGF21 protein in Asian seabass and human. The reliability of prediction was shown with different colors. Blue denoted with the highest level of reliability, while red denoted the lowest level of reliability. (TIF) [file pone.0090172.s001.tif]

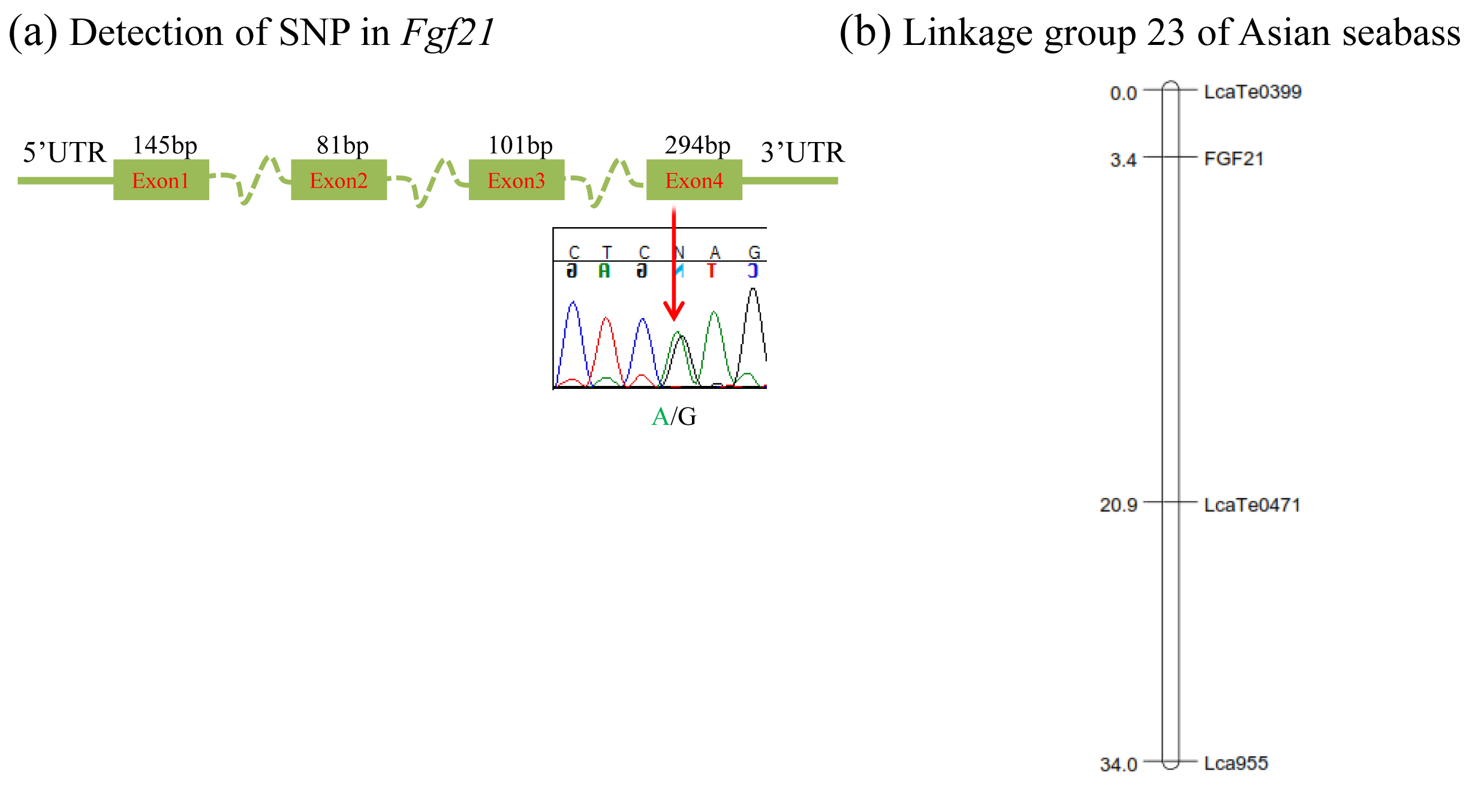

Supplement: Figure S2 — Single nucleotide polymorphism in the Fgf21 gene (a) and mapping of the Fgf21 gene on linkage group 23 (LG23; b) of Asian seabass. (TIF) [file pone.0090172.s002.tif]
